# 

**Published:** 1859-12

**Authors:** 


### Books, &c., Received.

We have received, and will notice in the next number more particularly: "Parrish's Practical Pharmacy;" "Habershon on the Alimentary Canal;" "Flint on Diseases of the Heart," and "Gross's System of Surgery," from the Publishers, Blanchard & Lea, through Messrs. J. Richards & Co., of this city. We also acknowledge, with the same promise, "Alcohol, Its Place and Power," by James Miller, Professor of Surgery in the University of Edinburgh, and the "Use and Abuse of Tobacco," by John Lizars, late Prof. of Surgery to the Royal College of Surgeons, from Messrs. Lindsay & Blakiston, Philadelphia.

In addition to these, a number of pamphlets, monographs, &c., which we will notice at our earliest convenience.

### TO CORRESPONDENTS.

By a recent change in the management of the Journal, its whole business affairs will hereafter come under the supervision of the Senior Editor, and it is therefore reqnested that ALL Communications, whether relating so the Editorial, Subscription, Advertising or Financial Departments, thould be addressed to Jos. P. LOGAN, or Editors of Medical and Surgical Journal, Atlanta, Ga.

